# Efficacy and Safety of Zoledronic Acid and Pamidronate Disodium in the Treatment of Malignant Skeletal Metastasis: A Meta-Analysis

**DOI:** 10.1097/MD.0000000000001822

**Published:** 2015-10-23

**Authors:** Liqing Yang, Shuai Du

**Affiliations:** From the Department of Orthopedics, Shengjing Hospital of China Medical University, Shenyang, China.

## Abstract

Solid tumors frequently metastasize to bone. Two bisphosphonates have been investigated for bone metastases including pamidronate disodium and zoledronic acid.

By searching the PubMed, Embase, Wanfang, and China National Knowledge Infrastructure (CNKI) databases, we conducted a meta-analysis to determine the efficacy and safety of zoledronic acid compared with pamidronate disodium in reducing pain in patients with bone metastases.

Studies were pooled, and the relative risk (RR) and its corresponding 95 % confidence interval (CI) were calculated. Version 12.0 STATA software was used for statistical analysis. Twenty relevant articles were included for this meta-analysis study.

The complete response rate in cancer patients treatment with zoledronic acid was significantly higher than that with pamidronate disodium (relative risk [RR] = 1.32 [95% confidence interval (CI), 1.00–1.75]; *P* = 0.987, *I*^*2*^ = 0%). However, there was no significant difference in the rate of partial response rate (RR = 1.04, 95% CI: 0.90–1.20; *P* = 0.942, *I*^*2*^ = 0%) and in the total effective rate (RR = 1.06, 95% CI: 1.00–1.12; *P* = 0.998, *I*^*2*^ = 0%). For adverse events (AE), the incidence of headache in cancer patients with zoledronic acid was significantly lower than that with pamidronate disodium (RR = 0.82, 95% CI: 0.70–0.96; *P* = 0.793, *I*^*2*^ = 0%). There was no significant difference in nausea or vomiting (RR = 1.00, 95% CI: 0.92–1.09; *P* = 0.494, *I*^*2*^ = 0%), fever (RR = 0.98, 95% CI: 0.85–1.14; *P* = 0.633, *I*^*2*^ = 0%), fatigue (RR = 1.01, 95% CI: 0.91–1.11; *P* = 0.914, *I*^*2*^ = 0%) and anorexia (RR = 1.31, 95% CI: 0.91–1.87; *P* = 0.024, *I*^*2*^ = 64.4%).

In conclusion, this meta-analysis indicates that treatment with zoledronic acid was more effective than pamidronate disodium in the complete response assessments and the incidence of headache, an AE, was significantly lower in cancer patients with zoledronic acid.

## INTRODUCTION

The skeleton is the third most common site to be invaded by metastatic cancer and the location of disease, resulting in the greatest morbidity. Every year, >1,500,000 patients will be affected by skeletal metastases in the world.^1^ Advanced cancer patients with bone metastases are common, ∼100% of myeloma patients, 70% to 80% of prostate or breast cancer patients and 30% to 40% of lung cancer or other solid tumors.^2^ In patients with bone metastases there is an increased risk of skeletal-related events (SREs) such as bone pain, pathologic fractures, impaired mobility, spinal cord compression, surgery or palliative radiotherapy, hypercalcemia.^3^ These SREs can limit patients’ functional independence and undermine their quality of life and place a heavy burden on affected patients and on the healthcare system.^4^

The main mechanism of bone metastasis is the activation of osteoclasts and bone-derived growth factor, which is induced by tumor cells to stimulate tumor growth.^5^ Current treatment of bone metastases in patients includes surgery, radiation therapy, bisphosphonate drugs, and painkillers, in addition to standard anticancer therapies.^6^ The main goal of treatment is to reduce the incidence of bone pain and improve quality of life and mobility. Bisphosphonates are osteoclast-mediated bone resorption potent inhibitor. Bisphosphonates has adopted a number of clinical trials in osteoclast activation of diseases, such as osteoporosis, hypercalcemia of malignancy, Paget's disease, and bone metastases.^7^ Pamidronate was approved by the US FDA in 1996 for treatment of skeletal metastases resulting from breast cancer and multiple myeloma (MM), and for Paget's disease of bone, and hypercalcemia of malignancy.^8^ Zoledronic acid was approved in 2002 in the United States for treatment of skeletal metastases caused by MM and solid tumors including lung cancer, prostate cancer, metastatic breast cancer (MBC), renal cancer, and colorectal cancer among others, after the benefits were noted.^8^ In this study, we performed a meta-analysis to assess the efficacy and safety of zoledronic acid and pamidronate disodium among patients with metastatic bone disease.

## MATERIALS AND METHODS

### Search Strategy

We searched for relevant studies through the PubMed, Embase, Wanfang, China National Knowledge Infrastructure Platform (CNKI) database (from January 1995 to March 2015) with the following terms and their combinations: “bisphosphonates/zoledronic acid/pamidronate disodium”, and “skeletal metastases/bone metastases”. All scanned abstracts, studies, and citations were independently reviewed. Disagreements were solved by full discussion until a consensus was reached. Moreover, references of the retrieved manuscripts were also manually cross-searched for further relevant publications.

### Selection Criteria

Controlled clinical trials evaluating the efficacy of zoledronic acid and pamidronate disodium for the treatment of cancer patients with skeletal metastases were included, if they met the following criteria: (1) controlled clinical trials should report either the effect estimates, such as RRs with 95% CIs, or sufficient information to calculate these values; (2) report of at least one of the following outcomes: (a) the complete response rate, (b) the partial response rate, (c) the total effective rate, or (d) adverse events (AE).

### Data Extraction

All the available data were extracted from each study by 2 investigators independently according to the inclusion criteria listed above. Primary outcomes were: (1) the complete response rate; (2) the partial response rate; and (3) the total effective rate. Secondary outcomes included: adverse events (AE)—(1) nausea or vomiting; (2) fever; (3) fatigue; (4) headache; (5) anorexia.

### Statistical Analysis

All statistical analyses were performed using the STATA v12.0 software (STATA Corp, College Station, TX). Dichotomous outcomes included rates or proportions were measured by relative risk (RR) and 95% confidence intervals (CI). When there was no significant heterogeneity (*P* >0.10), the fixed-effects model was used; otherwise, the random-effects model was used. Publication bias was evaluated by Begg's funnel plot and Egger's test and significance was set at *P* < 0.05 level.

## RESULTS

### Characteristics of the Studies

**FIGURE 1:**
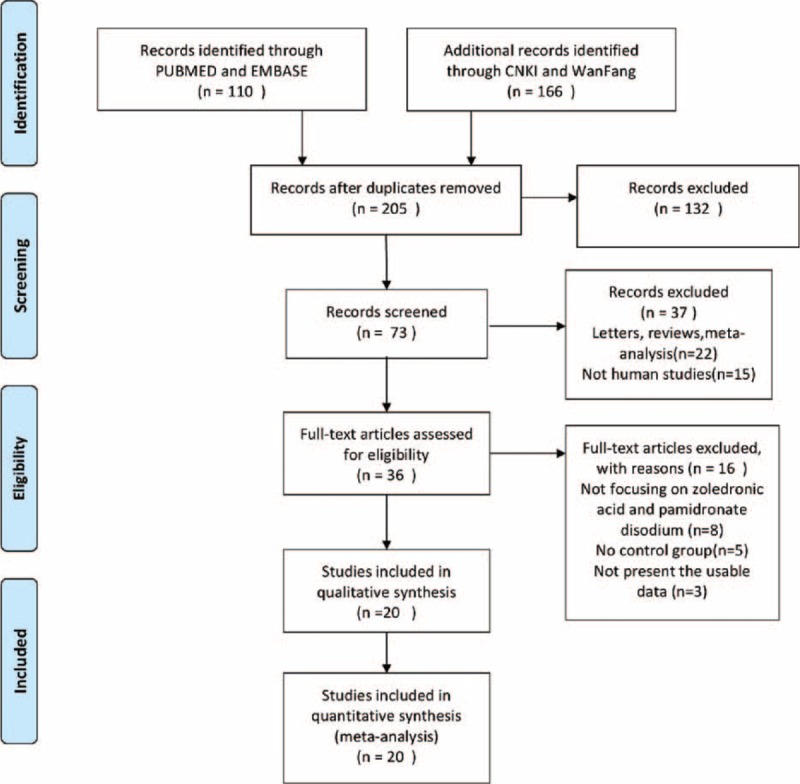
Flow diagram of studies identification.

There were 276 papers relevant to the search words. Subsequently, 203 irrelevant articles were excluded. The remaining articles were systematically reviewed, and all 36 articles qualified for full-text reading. After full-text reading, 16 articles were deemed unsuitable and were therefore excluded, and 20 articles were identified to be included for qualitative analysis. Finally, 20 controlled clinical trials were incorporated into the current meta-analysis.^9–28^ The flowchart of selection of studies and reasons for exclusion is presented in Figure 1.

### Quantitative Synthesis

All 20 studies including 3025 patients explored the efficacy and safety of zoledronic acid and pamidronate disodium in the treatment of malignant skeletal metastasis.

**FIGURE 2:**
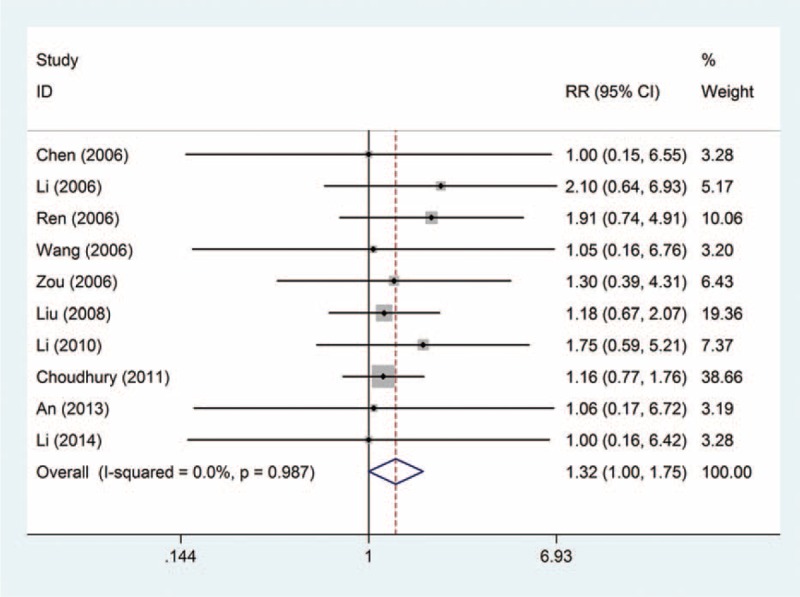
Relative risk (RR) and 95% confidence interval (CI) of individual studies and pooled data for the complete response rate in patients with bone metastases treatment with zoledronic acid compared with pamidronate disodium. CI = confidence interval, RR = relative risk.

The complete response rate: this outcome was reported in 10 trials, all comparing zoledronic acid to pamidronate disodium. There were 735 cases of patients, 376 cases in the treatment group, 359 cases in the control group. The heterogeneity was not statistically significant (*P* = 0.987, *I*^2^ = 0%), and the fixed effect model was used. The difference in the complete response rate was significant (RR = 1.32, 95% CI: 1.00–1.75), as shown in Figure 2.

**FIGURE 3:**
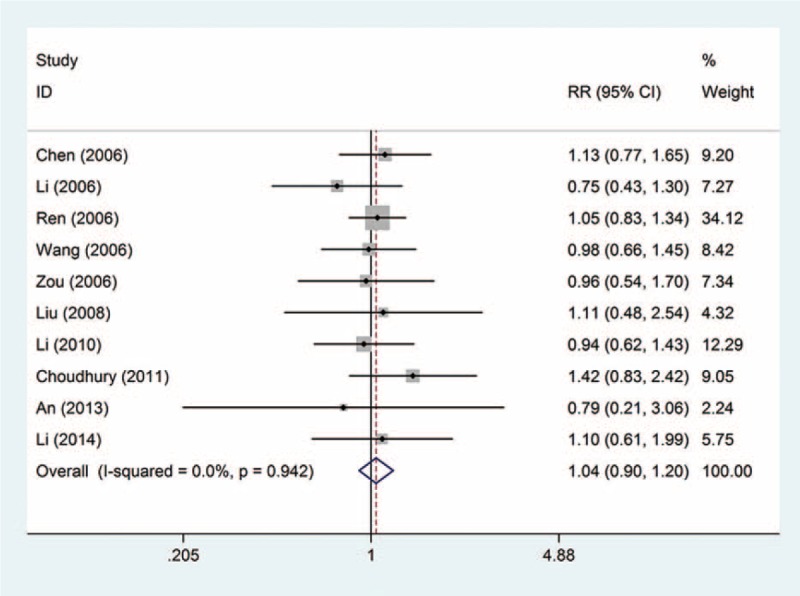
Forest plot of the relative risk (RR) and 95% confidence intervals (CIs) of studies on the partial response rate in patients with bone metastases. CI = confidence interval, RR = relative risk.

The partial response rate: this outcome was reported in 10 trials, all comparing zoledronic acid to pamidronate disodium. There were 735 cases of patients, 376 cases in the treatment group, 359 cases in the control group, the heterogeneity was not statistically significant, and the fixed effect model was used (*P* = 0.942, *I*^2^ = 0%). However, there was no significant difference in the rate of partial response rate (RR = 1.04, 95% CI: 0.90–1.20); see Figure 3.

**FIGURE 4:**
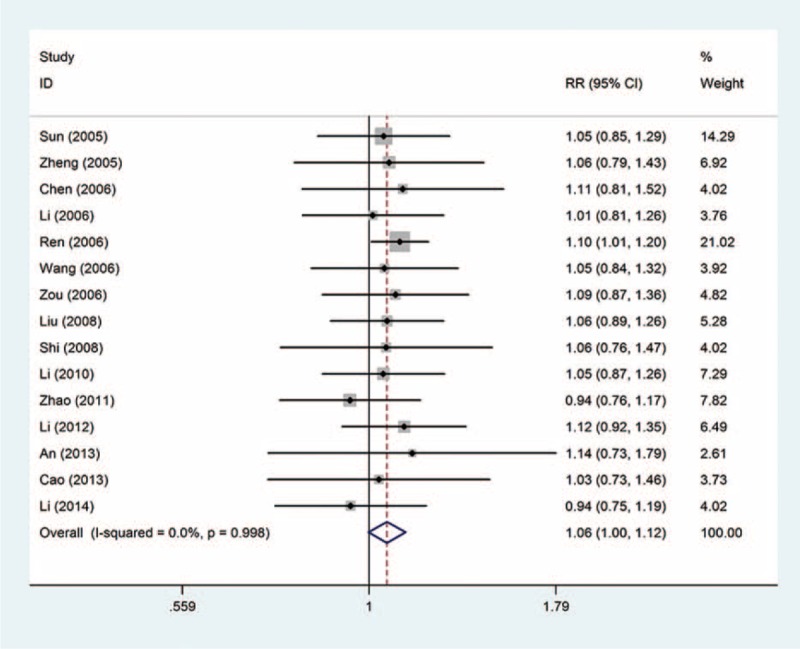
Forest plot of the relative risk (RR) and 95% confidence intervals (CIs) of studies on the total effective rate in patients with bone metastases. CI = confidence interval, RR = relative risk.

The total effective rate: this outcome was reported in 15 trials, all comparing zoledronic acid to pamidronate disodium. There were 1176 cases of patients, 609 cases in the treatment group, 567 cases in the control group, the heterogeneity was not statistically significant, and the fixed effect model was used (*P* = 0.998, *I*^2^ = 0%). However, there is no significant difference in the total effective rate (RR = 1.06, 95% CI: 1.00–1.12); see Figure 4.

**FIGURE 5:**
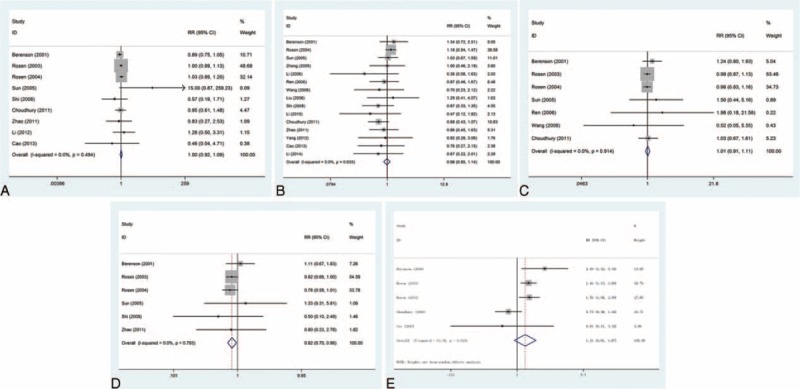
Forest plot of the relative risk (RR) and 95% confidence intervals (CIs) of studies on the incidence of adverse events (AE) in patients with bone metastases treatment with zoledronic acid compared with pamidronate disodium. (A) Nausea or vomiting; (B) fever; (C) fatigue; (D) headache; (E) anorexia. AE =  adverse events, CI = confidence interval, RR = relative risk.

Nausea or vomiting: this outcome was reported in 9 trials, all comparing zoledronic acid to pamidronate disodium. A total of 2256 patients were enrolled, 1124 patients in the treatment group, 1132 cases in the control group, there was no heterogeneity between the study, using the fixed effect model, and the results were (*P* = 0.494, *I*^2^ = 0%). However, there was no significant difference in the incidence of nausea or vomiting (RR = 1.00, 95% CI: 0.92–1.09), as shown in Figure 5A.

Fever: this outcome was reported in 15 trials, all comparing zoledronic acid to pamidronate disodium. There were 2043 cases of patients, 1022 cases in the treatment group, 1021 cases in the control group, the heterogeneity was not statistically significant, and the fixed effect model was used (*P* = 0.633, *I*^2^ = 0%). But there was no significant difference in the incidence of fever (RR = 0.98, 95% CI: 0.85–1.14), as shown in Figure 5B.

Fatigue: this outcome was reported in 7 trials, all comparing zoledronic acid to pamidronate disodium. There were 2259 cases of patients, 1117 cases in the treatment group, 1142 cases in the control group, the heterogeneity was not statistically significant, and the fixed effect model was used (*P* = 0.914, *I*^2^ = 0%). But there was no significant difference in the incidence of fatigue (RR = 1.01, 95% CI: 0.91–1.11); see Figure 5C.

Headache: this outcome was reported in 6 trials, all comparing zoledronic acid to pamidronate disodium. There were 2012 cases of patients, 992 cases in the treatment group, 1020 cases in the control group, the heterogeneity was not statistically significant, and the fixed effect model was used (*P* = 0.793, *I*^2^ = 0%). The difference in the incidence of headache was significant (RR = 0.82, 95% CI: 0.70–0.96), as shown in Figure 5D.

Anorexia: this outcome was reported in 5 trials, all comparing zoledronic acid to pamidronate disodium. There were 1839 cases of patients, 905 cases in the treatment group, 934 cases in the control group, the heterogeneity was statistically significant, and the random effect model was used (*P* = 0.024, *I*^2^ = 64.4%). However, there was no significant difference in the incidence of vomiting (RR = 1.31, 95% CI: 0.91–1.87), as shown in Figure 5E.

### Publication Bias

**FIGURE 6:**
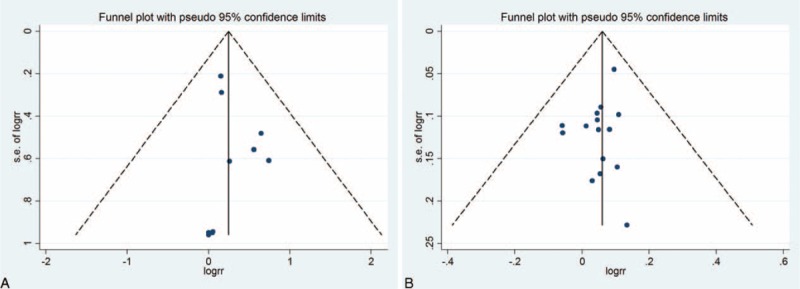
Begg's funnel plot for the publication bias test. Each point represents a separate study for the indicated association. (A) The complete response rate; (B) the total effective rate.

Finally, Egger's regression test showed no evidence of asymmetrical distribution in the funnel plot in the complete response rate (Begg's test *P* = 0.721; Egger's test *P* = 0.512) and the total effective rate (Begg's test *P* = 1.000; Egger's test *P* = 0.179) (Figure 6).

## DISCUSSION

There are reports of bone metastasis occur in ∼50% of patients with metastatic cancer, but some cancers, including breast, multiple myeloma, prostate, kidney, thyroid, and lung cancers are more frequently associated with clinical symptoms of bone disease.^29,30^ The growth of disseminated tumor metastases is a main cause of death in cancer patients. In the process of forming a primary lesion, tumor cells undergo a variety of molecular and epigenetic events that allow them to “escape” from the primary site of the tumor.^31^ The skeleton is a common metastasis site for high blood flow in the red marrow; the presence of tumor cell adhesive molecules that bind to stromal cells of the bone marrow; and these adhesive interactions lead to tumor cells, increase the yield of angiogenic factors and bone resorption factors, further enhancing the growth of bone tumors, thereby providing access to the resorbed bone matrix for subsequent tumor adhesion and proliferation.^32,33^ Skeletal metastasis is an important clinical problem that can cause other problems, such as fractures, severe pain, hypercalcemia spinal cord compression, and rapid degradation of the quality of life (QoL).

Bisphosphonates for bone-targeted drugs mainly inhibit osteoclast function and reduce bone resorption; other features include antitumor effects of bisphosphonate drugs^34^.The first-generation bisphosphonate drugs, such as clodronate and etidronate, have no nitrogen atoms in their structure as pyrophosphates analogs. They are metabolized to a cytotoxic ATP analog, adenosine-5-(β,γ-dichloromethylene)-triphosphate, thus inhibiting the mitochondrial adenine nucleotide translocase (ANT) and finally prompts apoptosis.^35,36^ The nitrogen atom is the part of bisphosphonates in an alkyl chain, such as pamidronate or alendronate, which are 10 to 100 times more effective than the first-generation bisphosphonate drugs and were demonstrated to control bone resorption by the mevalonate pathway.^37^ This pathway is essential for prenylation, which causes the delivery of either a geranylgeranyl or farnesyl moiety to targets such as Ras, Rac, Rho, or Rab, which are vital for cell morphology and cytoskeleton. No prenylation, the osteoclasts activity is reduced and apoptosis occurs.^38^ Some bisphosphonate drugs can be taken orally or intravenously and have been developed for bone loss and MBD. One of the most effective nitrogen-containing bisphosphonate drugs is the zoledronic acid (ZA), which is now widely used to decrease the incidence of SREs in MBD.

In this study, we conducted a meta-analysis to determine the efficacy and safety of zoledronic acid compared with pamidronate disodium in reducing pain in patients with bone metastases. Twenty relevant articles including 3025 patients were included for this meta-analysis study. We observed that the complete response rate in cancer patients treatment with zoledronic acid was significantly higher than that with pamidronate disodium (RR = 1.32, 95% CI: 1.00–1.75; *P* = 0.987, *I*^*2*^ = 0%). However, there was no significant difference in the rate of partial response rate and in the total effective rate. For AE, the incidence of headache in cancer patients with zoledronic acid was significantly lower than that with pamidronate disodium (RR = 0.82, 95% CI: 0.70–0.96; *P* = 0.793, *I*^*2*^ = 0%). There was no significant difference in nausea or vomiting, fever, fatigue, and anorexia. Zoledronic acid is a kind of the third-generation bisphosphonate. Its core bisphosphonate group attaches to bone and its imidazole ring gives its effectiveness. Zoledronate is the most effective bisphosphonate drug, and has around 100 to 1000 times the efficacy of pamidronate in vitro systems.^22^

Several limitations of our meta-analysis should be addressed. First, language can also introduce a bias. Specifically, we only choose either English or Chinese language or rule out other qualified research. Second, there is potential publication bias in this study as we did not take some unpublished papers and abstracts and considering their data was not available to us. A third potential limitation is that the number of studies for some parameter analysis was small which might lessen the statistical power. Despite the above limitations, this is the first example of a meta-analysis on the efficacy and safety of zoledronic acid compared with pamidronate disodium in reducing pain in patients with bone metastases. With the application of a statistical approach to combine the results from multiple studies in our meta-analysis and to achieve strong objectivity, all the research methods were carried out with strict inclusion and exclusion criteria, indicating the validity and significance of our conclusion.

In conclusion, despite the limitations of this meta-analysis, our study confirmed that treatment with zoledronic acid was more effective than pamidronate disodium in the complete response assessments and the incidence of headache, an AE, was significantly lower in cancer patients with zoledronic acid. Further studies with larger data set and well-designed models are required to validate our findings.
